# Transhiatal esophagectomy with gastric pull-up, pyloric exclusion and Roux-en-Y gastroenterostomy for the management of esophageal caustic injury

**DOI:** 10.1016/j.ijscr.2019.02.006

**Published:** 2019-02-13

**Authors:** Edson Gonçalves Ferreira Junior, Philippos Apolinario Costa, Larissa Melo Freire Golveia Silveira, Nayane Carolina Pertile Salvioni, Bruna Menon Loureiro, Sandra Lúcia Lodi Peres, Thiago Jardim Pereira

**Affiliations:** Universidade Federal do Vale do São Francisco, Av. José de Sá Maniçoba, S/N - Centro CEP: 56304-917, Petrolina, PE, Brazil

**Keywords:** Caustic ingestion, Esophagectomy, Gastric pull-up, Roux-en-Y gastroenterostomy

## Abstract

**Introduction:**

Ingestion of caustic materials can lead to digestive tube perforation involving the mouth, pharynx, esophagus and stomach (Vezakis et al., 2016 [1]). In this case report, the authors opted for gastric pull-up in a case of esophageal and pyloric stenosis secondary to caustic ingestion, and a Roux-en-Y gastroenterostomy in the lower portion of the gastric pull-up.

**Presentation of case:**

A 37 years-old male presented complaints of dysphagia, which had started 28 days before admission after the ingestion of a caustic liquid. An esophagogastroduodenoscopy was performed, and showed a complete occlusion of the esophagus, without the possibility of performing an esophagus dilatation or placing a nasoenteric tube. The option was made for a transhiatal esophagectomy with gastric pull-up, pyloric exclusion and Roux-en-Y gastroenterostomy. The patient was later admitted with a stenosis of the esophageal anastomosis, which was resolved after performing endoscopic dilatation.

**Discussion:**

The medical team opted to use the stomach for the reconstruction of the gastrointestinal transit due to less morbidity during manipulation of that organ, as well as safer anastomosis, when compared to the colon. In this case report, the esophagus and pylorus were generally compromised, however, with no apparent damage whatsoever in the stomach. Therefore, we opted to resect the esophagus and used the stomach to perform a gastric pull-up with the exclusion of the pylorus and reconstruction with a Roux-en-Y gastroenterostomy.

**Conclusion:**

The proposed surgery is an option when dealing with similar cases, where endoscopic dilatation is not an option, and there is an associated pyloric stenosis.

## Highlights

• The authors present a surgical option for the management of esophageal caustic injury.
• The surgery consists of a transhiatal esophagectomy with gastric pull-up, pyloric exclusion and Roux-en-Y gastroenterostomy.
• The technique can be indicated when esophagectomy is necessary and there is pyloric stenosis associated.

## 1 Introduction

Ingestion of caustic substances, besides being a medical emergency with high morbidity and mortality, is also an important cause of sequelae [1]. Its effects may range from necrosis [2] to perforation in the digestive tract, which may involve the mouth, pharynx, esophagus and stomach [1].

These lesions are found in children and adults, however, the etiology differs between accidental and intentional suicide attempts, respectively [[1], [2], [3], [4], [5], [6]]. Therefore, in children the lesions tend to be less severe, since the ingested volume is lower than the volume ingested by adults in an attempted suicide or homicide [1].

In those patients that survive, between the second and third week after the initial trauma, there may be complications such as tracheobronchial injury, necrosis and fistulas [1]. However, over the long term, complications may include esophageal stricture, dysphagia, and increased risk of esophageal cancer, which affects the patients' quality of life [1]. Thus, it is clear the importance of early diagnosis and effective therapy, since these does not only alter mortality, but also the morbidity of sequelae [1].

Among the treatment proposals for caustic stenosis are endoscopic dilatations and surgeries, for example the replacement of damaged portions with transverse colon [[7], [8], [9]]. In this case report, the medical team reports the option for gastric pull-up in a case of esophageal and pyloric stenosis, opting to perform a Roux-en-Y gastroenterotomy in the lower portion of the gastric conduit. This work was reported in line with the SCARE criteria [10].

## 2 Presentation of case

A 37 years-old caucasian male, with past medical history of paranoid schizophrenia, associated marijuana use presented to our service complaining of progressive dysphagia, that limits his intake of liquids, secondary to a lye (sodium hydroxide) ingestion 28 days prior to admission. The patient underwent an esophagogastroduodenoscopy, which showed complete occlusion of esophageal lumen, 22 cm from the incisors, with no possibility of dilation or nasoenteral tube passage. Barium esophagogram showed no contrast medium passage.

**Fig. 1:**
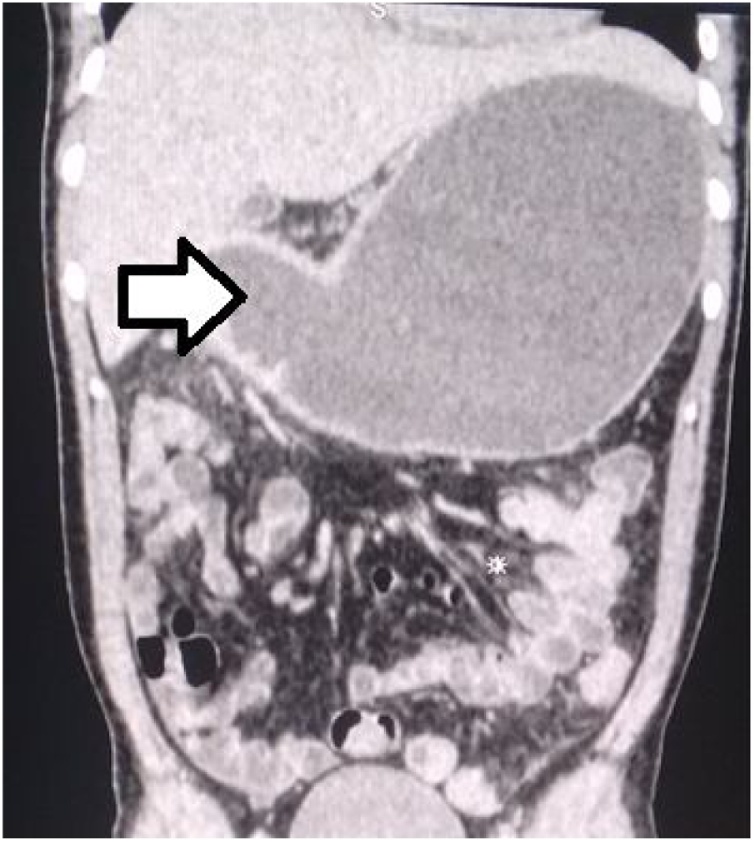
Computed tomography scan with contrast: The arrow points to the stomach that shows a severe dilatation, suggesting an associated pyloric stenosis.

A computer tomography of the abdomen revealed a severe dilatation of the stomach, suggesting an associated pyloric stenosis (Fig. 1).

**Fig. 2:**
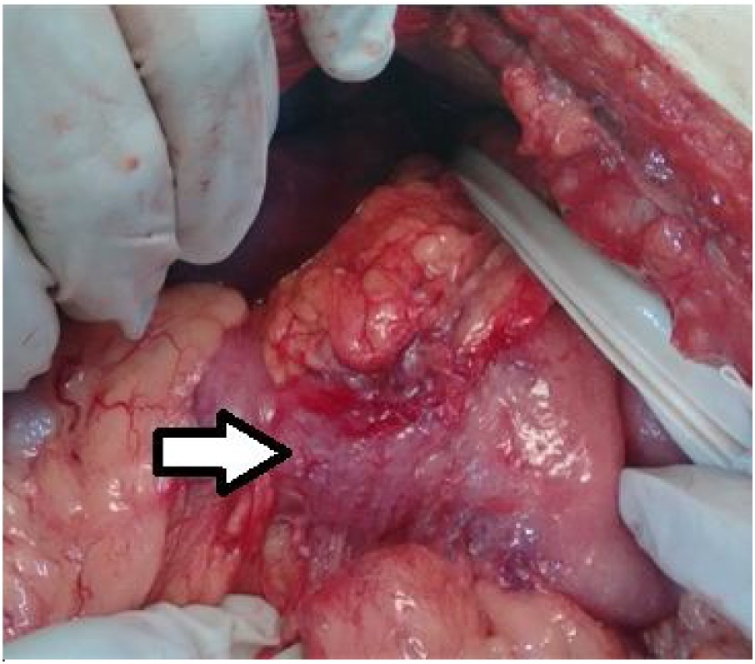
Intraoperative findings: The arrow points to the pylorus that shows stenosis.

**Figs. 3 and 4:**
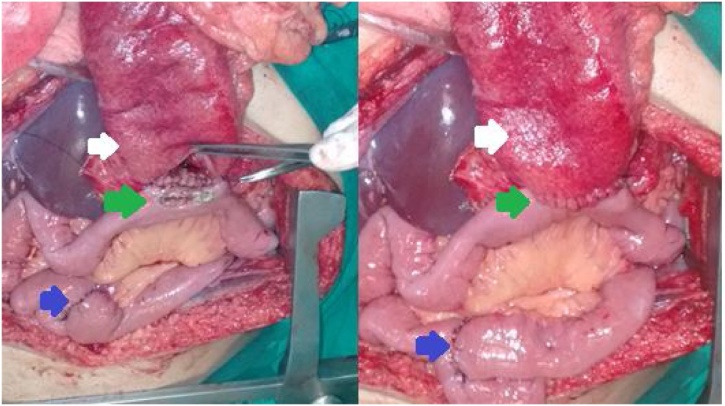
Intraoperative findings: Confection of gastric pull-up (white arrow) associated gastroenterostomy (green arrow) and enteroenterostomy (blue arrow) by a Roux-en-Y.

The medical team initially chose a transhiatal esophagectomy, along with reconstruction of the gastrointestinal tract with the colon, however, during the intraoperative period, only pyloric stenosis was observed with preservation of other portions of the stomach (Fig. 2). In light of the aforementioned, the team opted for confection of the gastric pull-up and cervical anastomosis associated with classic pyloric exclusion and Roux-en-Y gastroenterostomy (Figs. 3 and 4, Figs. 3 and 4). We performed a 6 h open procedure with no intraoperative complications or need of blood transfusions.

During the postoperative period, the patient developed a cervical fistula, with spontaneous resolution, and a retroperitoneal abscess treated with percutaneous drainage and antibiotic therapy. The patient was posteriorly discharged with an oral diet without restrictions.

One month after the operation, the patient presented with dysphagia. Upper endoscopy showed a cervical esophagogastric anastomosis stenosis that was resolved with endoscopic dilations. On the second year of follow-up, the patient had no dysphagia or any other symptoms of the disease.

## 3 Discussion

Caustic ingestion as an attempted suicide has an unknown prevalence [1]. The profile of these patients is usually young adults [2,4] and morbidity and mortality are estimated at rates above 20% [1,2]. The presence of symptoms and their characteristics vary according to the severity of organ involvement, time of ingestion, type of substance and etiology of the lesions [1], ranging from oligosymptomatic cases to dramatic cases with perforation of organs and peritonitis [3].

Endoscopy is the gold standard for the diagnosis of the lesions and should be performed in documented or suspected cases of corrosive substance intake, ideally within the first 24 h [1,3]. This procedure is capable of revealing the extent and severity of the lesions, directing the management of the case, as well as predicts morbidity and complications [1,3].

The most important complication in the late phase of caustic ingestion is the formation of stenosis, reported at rates close to 100% for transmural lesions [1]. In general, depending on the amount of substance ingested, stenosis can affect distal regions such as pylorus [1]. The stenoses can cause dysphagia and gastric obstructions, requiring multiple endoscopic or surgical approaches [1,6].

Also esophagectomy should be considered during the restoration of the alimentary pathway in selected cases [1]. There is no consensus in the literature concerning this procedure, but since the risk of developing esophageal neoplasia is estimated at 8% in 25–50 years, it is preferable to perform organ resection in younger patients, and avoid the procedure in elderly and with middle aged patients, except in cases where chest exploration is mandatory [1,6].

Restoration of intestinal transit is usually performed with transposition of a normal intestinal segment, and there is no definition of the best possible option, stomach or colon [4]. The first one has the advantages of good vascularization and the need of fewer anastomoses, and it has been recommended as the first option by several studies [[7], [8], [9]]. The colon can be used in cases of involvement of the stomach and duodenum; and has shown good results in places that consider it as the first option to replace the affected segment [1,5]. Transposition by the posterior mediastinum has a shorter route and a better functional outcome [1].

The medical team herein considers the stomach as the first option in our service, due to the lower morbidity in terms of organ manipulation and safer anastomoses, when compared to the colon. Also, when using certain segments of the colon, there might be the need for vascular evaluation with a contrasted study.

In this case report, the impairment was restricted to the entire extension of the esophagus and pylorus, as seen from the attached figures. There was no possibility of passage for a dilator that would allow endoscopic treatment. However, the antrum, body and gastric fundus did not present alterations. Thus, the team chose to resect the esophagus due to the age of the patient through a transhiatal approach, avoiding manipulation of the thorax. We also used the stomach for the manufacture of the gastric pull-up and associated the procedure with a pyloric exclusion and reconstruction of the transit with Roux-en-Y gastroenterostomy.

Complications concerning this particular case were a cavitary abscess, a fistula and a cervical anastomosis stenosis, which were treated by ultrasound guided drainage, conservative clinical management and upper endoscopy with dilatation of the anastomosis, respectively. All these complications are also present in the other surgical options with a variable incidence due to heterogeneity of surgical techniques and disease like malignancy, achalasia or stenosis. The most common are pulmonary complications, 22%, stenosis 8% and leak 5% [11].

The number of anastomosis needed for reconstruction of the tract is equal using the stomach or the colon, but with the stomach theoretically would be less morbid due to an abundant and safe vascularization of that organ, thus there is no need for a preoperative angiographic study.

## 4 Conclusion

The proposed surgery may be an option in cases where endoscopic dilation is not an option and there is association with pyloric stenosis, given the discussed disadvantages of an esophagogastrectomy with neoesophagus confection by colon apposition. To assess which strategy is superior, and in which cases their indication is better placed, there is left the need of future evaluations with more extensive studies.

## Conflicts of interest

None.

## Funding

Authors did not receive any funding for this work.

## Ethical approval

We do not require ethical approval to write a case report paper.

## Consent

Written informed consent was obtained from the patient’s family member for publication of this case report and accompanying images. A copy of the written consent is available for review by the Editor-in-Chief of this journal upon request.

## Author contribution

Edson Gonçalves Ferreira Junior: Operated the patient, Conceptualization, Methodology, Resources, Writing the paper, Writing – Review & Editing, Project Administration, Final approval.

Philippos Apolinario Costa: Conceptualization, Methodology, Data collection, Data analysis/interpretation, Writing – Review & Editing, Final approval.

Larissa de Melo Freire Gouveia Silveira: Operated the patient, Conceptualization, Methodology, Data collection, Resources, Writing – Review & Editing, Final approval.

Nayane Carolina Pertile Salvioni: Conceptualization, Methodology, Data collection, Resources, Writing – Review & Editing, Final approval.

Bruna Menon Loureiro: Conceptualization, Methodology, Data collection, Resources, Writing – Review & Editing, Final approval.

Sandra Lúcia Lodi Peres: Operated the patient, Conceptualization, Methodology, Data collection, Resources, Writing – Review & Editing, Final approval.

Thiago Jardim Pereira: Operated the patient, Conceptualization, Methodology, Data collection, Resources, Writing – Review & Editing, Final approval.

## Registration of research studies

Case reports don’t need to be registered.

## Guarantor

Edson Gonçalves Ferreira Junior.

## Provenance and peer review

Not commissioned, externally peer-reviewed.
